# Development and evaluation of the Food Bank Health and Nutrition Assessment

**DOI:** 10.1017/S1368980023000204

**Published:** 2023-04

**Authors:** Cassandra J Nguyen, Caitlin Kownacki, Veronica Skaradzinski, Kaitlyn Streitmatter, Stephanie Acevedo, Stephen D Ericson, Jessica E Hager, Jennifer McCaffrey

**Affiliations:** 1 Nutrition Department, University of California, Davis, Davis, CA, USA; 2 Office of Extension and Outreach, University of Illinois at Urbana-Champaign, Urbana, IL, USA; 3 Feeding Illinois, Geneva, IL, USA; 4 Feeding America, Chicago, IL, USA

**Keywords:** Charitable food assistance, Food insecurity, Community nutrition, Nutrition environment, Policy, systems and environment, Public health, Health equity

## Abstract

**Objective:**

Efforts to improve the nutritional quality and health promotion in the charitable food system have been undertaken. Though methods exist to track these efforts in terms of food banks’ inventory, there are not research-tested tools to assess, monitor and influence policy, systems and environmental (PSE) changes. The study objective was to develop and evaluate a novel assessment tool that could be used to evaluate a food bank’s efforts to improve the promotion of health and nutritious foods.

**Design:**

The study had five phases: (1) initial development; (2) iterative review and revisions; (3) pilot testing; (4) content validity assessment and (5) inter-rater and test-retest assessment. The Food Bank Health and Nutrition Assessment (FB-HANA) was drafted after reviewing existing policies, nutrition-focused charitable food systems guidance and similar tools for food pantries.

**Setting:**

Midwestern United States.

**Participants:**

Eleven food banks.

**Results:**

Stakeholders and pilot testers provided initial feedback to refine the FB-HANA’s flow, ease of completion and collection of contextual information. External experts rated the FB-HANA and each of the eight objectives as content valid. A set of two assessments completed by twenty-six community-based professionals, employed by Extension and fourteen food bank staff across eleven food banks, supported moderate to excellent inter-rater and test-retest reliability for the FB-HANA overall and each of its objectives.

**Conclusions:**

Evidence suggests that the FB-HANA can be used by either food bank or community-based professionals, such as Extension staff, to provide a perspective on ways food banks promote health and nutrition through PSE approaches.

Attaining and maintaining a healthy diet is considerably difficult for the millions of people in the USA who experience food insecurity, defined by the US Department of Agriculture as an economic and social condition of limited or uncertain access to adequate food^(1)^. The likelihood of experiencing food insecurity is unevenly distributed across the population. As a product of historical and modern policies and practices, African American, Latino and Native American individuals experience food insecurity at higher rates than non-Hispanic white adults^(1,2)^. The US government has several efforts, such as the Supplemental Nutrition Assistance Program (SNAP)^(3)^, which attempt to address food insecurity. However, some households experiencing food insecurity do not meet eligibility criteria for federal programs, or for those who do, the benefits are at times insufficient to address the households’ food needs^(4)^. In response to these gaps in the social safety net, non-governmental private organisations – broadly termed the charitable food system – have arisen to distribute foods to individuals with unmet needs.

The charitable food system in the USA is composed of organisations with storage warehouses (i.e. food banks) that often accept all types and sizes of food donations, purchase large quantities of food and receive food from federal programs. Food banks then re-distribute the food through consumer-facing sites (e.g. food pantries, shelves, soup kitchens, programs, etc.). Consumer-facing sites then make groceries or hot meals available to individuals (commonly referred to as neighbours, guests or clients). This system serves individuals and households in immediate need of food, often with minimal or no screening requirements, and is increasingly providing additional services (e.g. SNAP application assistance, referral to community health services) aimed at addressing systemic and longer term needs^(5)^.

Developed largely in the 1980s in the USA, this system was originally referred to as the ‘emergency food system’ to reflect the short-term supply of food distributed to households^(6)^. However, there is increasing evidence of long-term use among food pantry clientele^(7,8)^. Today, the largest non-profit network of food banks in the USA, Feeding America, includes 200 food banks and 60 000 food pantries and meal programs across the country to support the needs of individuals and families^(9)^. An estimated 20 % of households in a nationally representative sample were served by the charitable food system in 2020^(10)^. Given that the system serves individuals at high risk of food insecurity^(7,11,12)^ and provides a non-negligible portion of users’ diets^(13–15)^, the charitable food system is an opportune setting for nutrition-related programming, as well as efforts to meet individuals’ cultural food needs and preferences.

There are notable critiques of the charitable food system’s historical and, in some cases, current efforts to provide services that promote food security and nutrition. Assessments of food pantry inventories have indicated shortfalls of key nutrients and food groups^(16)^. However, it is important to note this low quality of foods may be a reflection of the broader US food system and supply priorities, and a by-product of the charitable food system’s reliance on donated foods. Though actors in the charitable food system increasingly recognise health promotion, equity and nutrition as important – if not vital – parts of a food bank’s role, published estimates suggest a minority of food banks have formal nutrition policies^(17)^. Nutrition policies are one tool for food banks to develop decision markers for food donations, purchasing or data collection.

Food banks and food pantries face the challenge of persistent variability in the quantity, quality and type of foods received through donations. This variability has consequences for the staff and volunteers needed to handle and process donations^(18)^. Stakeholders have noted issues of safety and appeal of perishable foods received for distribution, which may be considered inedible and necessitate disposal by food banks, pantries or clients^(19)^. Cultural relevance and usability of items distributed are also of importance. Surveys of clients have indicated that some food pantries do not have food their families like, or they do not know how to prepare the available foods^(19–21)^.

Despite the barriers, there are many ongoing efforts to improve the charitable food system’s policies, systems and environments (PSEs) with the aim of supporting individual and community nutrition and health. Much of the published work in this area has focused on initiatives in food pantries, as the consumer-facing side of the system. These initiatives have included efforts such as distribution of suggested donation lists^(22,23)^, coordination with food policy councils^(24,25)^, developing nutrition-focused policies, transitioning distribution from traditional pre-packaged boxes to client-choice models^(22–24)^, incorporating wrap-around social services^(24,26)^, partnering with the health care system^(27)^ and reorganising how items appear to ‘nudge’ clients towards healthier items^(22–24,28)^, among others^(22,23,26)^. These initiatives – many of which began within the last decade – focus on making healthier choices easy and accessible^(29)^ and are coupled with established nutrition education programming^(30)^.

Though not as widely documented in the literature, changes to practices and policies in food pantries have coincided with upstream initiatives undertaken by food banks^(31)^. In efforts to track and improve the nutritional quality of food banks’ inventories, a variety of nutrition ranking systems and guidelines were developed^(32–34)^. In 2019, a national panel of experts convened by Healthy Eating Research (HER; a national program of the Robert Wood Johnson Foundation) developed the HER Nutrition Guidelines for the Charitable Food System to promote consistent and approachable nutrition classifications for diverse charitable food settings across the USA.^(35)^ In addition to these national efforts to rank and shift the nutritional composition of food banks’ inventories, case studies have detailed how individual food banks have implemented incremental changes to policies and practices to focus on nutrition and well-being of their community members^(36,37)^. A food bank in New England modified the ordering system their food pantry agency members interacted with and found that the introduction of nutrition ranking information to this system resulted in significant increases in the nutritional quality of monthly orders^(38)^. Another food bank in New York state assessed the optimal combinations of days and volunteers to increase the amount of fresh produce they could glean from local farmers to supplement their inventory^(39)^. As of 2021, 20 % of the food banks in the Feeding America network reported using the HER Nutrition Guidelines for the Charitable Food System (J. Hager, personal communication, May 4, 2022). Though the HER nutrition ranking system provides a method for food banks to assess the nutrition quality of their food inventory, there have been no published methods to capture the range of supplementary practices and policies that food banks have been using to promote nutrition and health.

The current study was undertaken to address the need for a method to assess, measure and track the different strategies used by food banks to promote health and nutrition through their practices and policies. This work was started by staff affiliated with the University of Illinois Extension who are responsible for implementing SNAP-Ed in the state of Illinois and who collaborate with food banks interested in updating policies and practices to better promote nutrition and health. The objective of the current study was to develop and evaluate a novel assessment tool that could be used by food bank staff and partnering community-based professionals, such as Extension staff, in diverse settings to evaluate efforts to improve the promotion of health and nutrition in food bank settings. The study was undertaken with the expectation that the final tool could be used by SNAP-Ed implementing agencies, such as Extension and other community-based health professionals, as well as food bank staff and volunteers.

## Methods

**Fig. 1:**
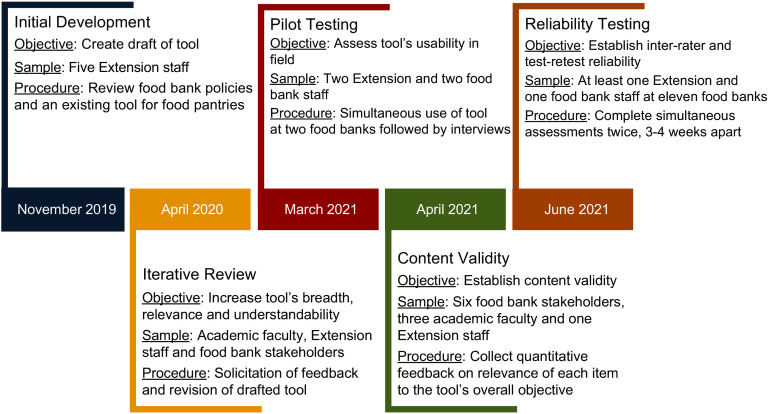
Five phases and timeline of food bank-health and nutrition assessment (FB-HANA) development and evaluation

The Food Bank Health and Nutrition Assessment (FB-HANA) was developed as a tool to quantify the ways food banks promote health and nutrition among charitable food recipients through PSE approaches. The goal of completing an FB-HANA is to gain new insights about health and nutrition practices, identify areas of opportunity for action planning and to determine a baseline against which progress can be measured. The FB-HANA was created for use as a self-assessment or as a measure that external assessors could use to support food banks. Assessments were completed with a combination of site observations, review of food bank’s documents and interviewing food bank staff (if completed by an external assessor). The development and evaluation of the FB-HANA was conducted over five phases (Fig. 1): (1) initial development; (2) iterative review and revisions; (3) pilot testing; (4) content validity assessment and (5) inter-rater and test-retest assessment. The distinct steps and the stakeholders involved in each phase are described below.

### Initial development

The development of the FB-HANA was initiated in November 2019 as a product of collaborative efforts between Extension staff and staff within a statewide charitable food distribution network to promote the adoption of nutrition policies by food banks in Illinois. To evaluate the effectiveness of these collaborative efforts to prompt nutrition policy adoption and implementation of related practices, staff searched for existing assessment tools. No tool was identified and staff at food banks and charitable food networks indicated their interest and need for such an assessment tool with the research team. Thus, the FB-HANA development team convened in March 2020 to create an assessment that met the needs of the IL-based nutrition policy initiative while also being usable by food banks in other states. The FB-HANA development team was composed of five Extension staff who had backgrounds in public health, social work and community nutrition and experience partnering with charitable food distribution organisations to promote nutrition and health. FB-HANA development team members generated an initial list of FB-HANA objectives and related strategies based on a review of food bank policies (five in Illinois and fifteen from other states) as well as adapted strategies from an existing assessment tool for food pantries^(22,23)^.

### Iterative review and revisions

To increase the breadth of the tool and ensure that it was written in terms that were relevant and understandable to a variety of audiences, the draft of the FB-HANA was sent to external stakeholders to provide feedback and suggested revisions. Stakeholders included academic faculty with expertise in community nutrition and evaluation; Extension staff with pragmatic experience working with food banks; staff at food banks that partnered with Extension and staff at charitable food distribution networks. Feedback was solicited and used to revise the draft of the FB-HANA from April 2020 to February 2021. During this phase, a faculty member with experience developing and evaluating tools to assess the nutrition environment in charitable foods organisations^(22)^ joined the FB-HANA development team.

### Pilot testing

The FB-HANA was pilot tested by Extension and food bank staff at two food banks to understand its usability in the field. One of the food banks was affiliated with Feeding America and the other food bank was affiliated with a regional food bank network. The selection of food banks in differing networks was intentionally done to determine usability in different food bank systems. The Extension and food bank staff completed the FB-HANA in-person together. Both the food bank and Extension staff were interviewed after each assessment to ask about their overall impressions of the process, challenges faced and any suggested changes to the FB-HANA. The interviewer took notes during each interview and compiled them into a summary. The summary was reviewed by the FB-HANA development team to inform additional revisions. The pilot testing phase was completed between March 2021 and April 2021.

### Content validity assessment

To establish content validity, invitations were sent to practitioners and academic faculty to provide a review of the FB-HANA. Potential reviewers were identified through an email listserv of charitable food staff, publications of food banking nutrition research and existing partnerships. Reviewers were sent an electronic questionnaire to assess content validity of the FB-HANA. On this questionnaire, each objective of the FB-HANA and the overall tool was scored according to its relevance to the objective ‘*To quantify ways food banks promote health and nutrition through policy, system and environmental approaches*’ on a four-point scale. The scale ranged from 1 = not relevant, 2 = unable to assess relevance without item revision or item is in need of such revision that it would no longer be relevant, 3 = relevant but needs minor alterations to 4 = extremely relevant. The questionnaire also included an open-answer item for reviewers to identify their background and experience with the charitable foods sector. Reviewers were asked to return their reviews within 2 weeks of being sent the questionnaire and eight individuals returned completed content validity assessments. Responses on the four-point scale were compared with content validity index thresholds and to be considered content valid at least 80 % of reviewers needed to rate each objective and the overall tool as a 3 or above on the four-point scale^(40)^. The content validity phase was completed between April and June 2021.

### Inter-rater and test-retest reliability assessment

Between June and October 2021, all food banks affiliated with Feeding America and Midwest Food Bank in Illinois were invited to participate in the reliability testing phase. Each food bank that agreed to participate in this phase was scheduled to complete two FB-HANAs, separated by 3–4 weeks. At least one Extension staff and at least one food bank staff independently completed FB-HANA documentation during each assessment. Extension staff had prior experience conducting assessments in other charitable food settings, but no training specific to the FB-HANA procedures were provided. All staff completing assessments were provided a link to the FB-HANA on the Qualtrics, LLC (Provo, UT) web-based platform 1 to 2 d in advance of the scheduled assessment. Staff were asked not to discuss content of the FB-HANA or the answers they selected with other staff. Responses were required for all scored components of the FB-HANA to ensure the reliability estimates were based on complete datasets. During each assessment, one additional Extension staff was present to serve as a ‘monitor’ to ensure that there was no discussion about selections made and any logistical questions were answered. Monitoring staff did not submit any data for the reliability estimates. The second assessment conducted 3–4 weeks after the initial assessment was completed by the same staff as the initial assessment.

Both reliability outcomes were estimated with intra-class correlation coefficients. Inter-rater reliability was calculated by comparing scores at the initial assessment produced by different staff. This comparison was made for overall FB-HANA scores and scores for each of the FB-HANA’s objectives. The intra-class correlation coefficient (ICC) for inter-rater reliability was produced from a one-way random-effects model. The one-way random-effects model was first generated based on all assessors present at the initial assessment. A second model was built based only on the Extension staff completing assessments at the initial assessment to compare the inter-rater reliability produced. Test-retest reliability was calculated by comparing FB-HANA overall scores and scores on each of the objectives produced at the two different assessments by the same staff members. The ICC for test-retest reliability was produced from a mixed effects model based on all assessors who completed both a first and second assessment at their respective food bank(s). The first model only included an ID variable for the food bank, whereas a second model was built that included a dummy variable indicating whether the assessor was an Extension staff. This second model was used to compare how assessments completed by food bank staff differed from those completed by Extension staff. Each ICC and the corresponding 95 % CI was interpreted as <0·5 = poor reliability, 0·5–0·75 = moderate reliability, 0·75–0·9 = good reliability and >0·9 = excellent reliability^(41)^.

## Results

### Initial development, iterative review and revisions

The FB-HANA development team produced a list of potential strategies and related objectives for the tool. This list was shared during meetings and via e-mail with seven stakeholders with experience working with the charitable foods system to provide feedback. The purpose of the tool was clarified during this phase, with the team deciding the goal of the assessment was to ‘provide perspective on ways food banks promote health and nutrition through policy, system and environmental approaches.’ The HER Nutrition Guidelines for the Charitable Food System were published during this phase^(35)^ and strategies focused on equity were added after the draft of the tool was compared against the guidelines. The preliminary list of strategies was organised into eight objectives (Table 1). Each scored strategy was structured as a statement which the assessor would answer yes or no to for ease of completion. To support programmatic planning, additional information about the food bank, such as participation in a formal network, special programs and reach, sources of food and funding and external conditions were collected (but not scored) on the FB-HANA.

**Table 1:** Objectives and strategies in the food bank health and nutrition assessment (FB-HANA)

Objective	Strategies#	Inter-rater reliability	95 % CI	Test-retest reliability ICC	95 % CI
Objective 1: Food bank identifies and responds to nutrition needs of communities served	6	0·95	0·75, 0·96	0·60	0·36, 0·80
Objective 2: Food bank integrates needs of diverse populations	6	0·89	0·51, 0·92	0·73	0·52, 0·88
Objective 3: Food bank adopts, implements and shares nutrition policy	13	0·99	0·93, 0·99	0·98	0·96, 0·99
Objective 4: Food bank provides nutrition education training and nutrition education resources	7	0·90	0·54, 0·92	0·71	0·48, 0·86
Objective 5: Food bank fosters a variety of external partnerships	7	0·90	0·54, 0·92	0·71	0·49, 0·87
Objective 6: Food bank prioritises health in internal operations and with member agencies	11	0·95	0·72, 0·96	0·82	0·64, 0·92
Objective 7: Food bank considers nutritional quality of commodity, purchased and donated foods	9	0·95	0·71, 0·96	0·78	0·58, 0·90
Objective 8: Food bank models nutrition promotion practices to member agencies	6	0·87	0·46, 0·90	0·77	0·57, 0·90
Overall: Provide perspective on ways food banks promote health and nutrition through policy, system and environmental approaches	65	0·91	0·59, 0·93	0·81	0·63, 0·91

Note. CI = confidence interval; ICC = intra-class correlation coefficient.

ICC estimates for inter-rater reliability were produced from one-way random-effects models based on 3–5 raters at 11 food banks.

ICC estimates for test-retest reliability were produced from mixed effects models based on two assessments completed by 3–5 raters at 11 food banks.

### Pilot testing

All Extension (*n* 2) and food bank (*n* 2) staff who participated in pilot testing provided feedback via interviews or e-mail correspondence. Overall, the FB-HANA was considered useful with good flow between sections and feasible to complete. Staff estimated the FB-HANA took approximately 70 min to complete but shared that almost half of this time was dedicated to finding the documents needed to provide accurate information on food and funding sources for unscored components of the FB-HANA. To improve efficiency of time used, suggestions to clarify what the assessment entails, who in the food bank should complete it and the documents which may be needed were incorporated into the cover page of the FB-HANA. Feedback led to additional clarification of terms, reasons for some data collection and an explicit acknowledgement on the FB-HANA cover page that not all strategies would be relevant for each food bank. At the end of the pilot test, the food bank staff indicated interest and eagerness to use the results to begin planning their next steps; given this, the FB-HANA was placed in a web-based surveying platform (Qualtrics), formatted for ease of completion on mobile or portable computing devices (i.e. tablets or laptops) and structured to auto-generate a report of results for users immediately upon completion.

### Content validity assessment

The eight content validity reviewers included four food bank staff, one Extension staff, and three academic researchers. All reviewers rated the FB- HANA overall and objectives 1–3 and 5–8 as relevant (3 or above on 4-point scale), far exceeding the 80 % threshold required. Objective 4 was rated as relevant by most reviewers (88 %), still meeting the necessary threshold for content validity. Qualitative comments from reviewers resulted in re-ordering of objectives and strategies as well as revisions to strategy and objective wording. As one example, a strategy focused on provision of microwave recipes was updated to more general phrasing that recipes provided ‘require minimal cooking equipment.’ Additionally, four new strategies were added to increase the number of relevant practices and policies assessed.

The final version of the FB-HANA was produced after incorporating content validity feedback. All sixty-five strategies were answered with binary response of options of ‘Yes, this strategy is present at the time of the assessment’ or ‘No, this strategy is not present at the time of the assessment.’ These response options are not sensitive to strategies which are in development or generally present, but not on the day of assessment. Yet, the restrictive binary option of yes or no was selected to maximise ease of use and reliability. The full FB-HANA is freely available online on a web platform which incorporates display logic^(42)^, and a list of the individual strategies is shown in a Supplemental Table. Of note, some strategies are organised as ‘sub-strategies’ and are displayed only if a preceding question was answered to indicate the ‘sub-strategies’ were relevant. For example, if the food bank indicates they do not have a written nutrition policy, the following twelve strategies under objective 3 are not displayed (and subsequently unaffirmed). Finally, the FB-HANA development team decided to incorporate a definition of nutritious into the instructions to create a more uniform understanding among assessors. If the food bank had a nutrition ranking system in place, this was to be used to inform the interpretation of the term nutritious used throughout the FB-HANA; if the food bank did not have a ranking system in place, items categorised as green by the HER guidelines^(35)^ were used as the reference for what was to be considered nutritious.

### Inter-rater and test-retest reliability assessment

All eleven food banks invited to participate agreed to contribute to the reliability assessment. Descriptive characteristics of these food banks based on food bank staffs’ selections for non-scored elements of the FB-HANA during the first assessment are shown in Table 2. A total of forty individuals participated as raters. There were twenty-six Extension staff serving as raters with a mean of 2·4 (0·5 sd) Extension staff completing ratings per food bank. There were fourteen food bank staff completing assessments, with a mean of 1·3 (0·5 sd) staff per food bank. Each food bank staff only assessed their own respective food bank, whereas Extension staff rated a range of 1–7 unique food banks (mean = 2·2 and sd = 2·1).

**Table 2:** Characteristics of food banks who participated in the inter-rater and test-retest reliability phase for the evaluation of the food bank health and nutrition assessment (FB-HANA)

Characteristic	Food banks (*n* 11)	
	%	*n*
State		
Illinois	64	7
Other	36	4
	Mean	sd
Reach of food bank		
Member agencies	260·6	190·0
Households served	194 710·2	246 293·1
	%	*n*
Part of a larger food bank network		
Yes	100	11
No	0	0
	Mean	sd
Percent of food bank’s inventory from different sources		
Federal commodities	21·7	19·5
Manufacturing	21·5	26·2
Private local donors (e.g. local faith-based groups, non-food retail businesses, individual donors, etc.)	20·3	18·3
Food retailers (e.g. local grocery stores)	21·8	27·2
Local farmers or growers	2·8	3·1
Another food bank	2·8	2·6
Other (gleaning group, mixed sources, etc.)	13·9	20·1
	%	*n*
Sources of funds used to purchase food		
Food industry corporations, foundations, partners	91 %	10
Non-food industry corporations, foundations, partners	100	11
Individual donors	100	11
Grants	100	11
Member agency fees	64	7
Other	36	4
Food bank tracks the amount of food items considered nutritious		
Yes	64	7
No	36	4
Special programs offered by food bank		
Child and Adult Care Food Program (CACFP)	36	4
Summer Food Service Program (SFSP)	27	3
USDA Commodities	46	5
School-based food pantries	82	9
Healthcare-based food pantry	82	9
Mobile food distributions	100	11
Supplemental Nutrition Assistance Program (SNAP) outreach	73	8
Disaster food assistance	73	8
Cooking education or demonstrations	55	6
Other (backpack program, college-based pantries, etc.)	73	8
	Mean	sd
Number of staff completing assessment		
Food bank staff	1·3	0·5
Extension staff	2·4	0·5

Note. Estimates rely on data provided by the food bank staff at the initial assessment. In the case that >1 food bank staff member was present at the initial assessment and responses were not identical, the last response recorded was used.

The inter-rater reliability of the overall FB-HANA among all raters met the threshold for excellent reliability (Table 1), and the inter-rater reliability for individual objective scores indicated good to excellent reliability. When ICC estimates for inter-rater reliability of the overall FB-HANA score were produced from a one-way random effects model restricted to the two to three Extension staff at the initial assessments, the ICC increased to 0·96 with a narrower 95 % CI of 0·80, 0·98 (data not shown). The second assessment was completed at all food banks 3 to 5 weeks (mean = 3·9, sd = 0·6) after the initial assessment. The test-retest reliability of the overall FB-HANA when comparing first and second assessments from all raters met criteria for excellent reliability (Table 1). Test-retest reliability for individual objectives varied from moderate to excellent reliability, with the lowest ICC produced for *Objective 1: Food Bank Identifies and Responds to Nutrition Needs of Communities Served* and the highest produced for *Objective 3: Food Bank Adopts, Implements and Shares Nutrition Policy*.

A second mixed-effects model with a dummy variable indicating affiliation as Extension or food bank added was built to compare how scores differed by assessor type. Extension staff produced significantly lower FB-HANA scores (*β* = −1·28; 95 % CI (−2·45, −0·11); *P* = 0·032) than their food bank staff counterpoints. Additionally, the ICC estimate of test-retest reliability was marginally higher (ICC = 0·82) with a narrower 95 % CI of 0·65, 0·92.

## Discussion

The objective of the current study was to develop and test a tool that could be used by food bank and community-based nutrition professionals to assess current practices to promote nutrition and health through PSE initiatives and monitor change over time. Findings indicate that the resulting tool, the FB-HANA has strong content validity as well as good to excellent inter-rater and test-retest reliability. Results indicate the FB-HANA can be employed directly by food banking professionals, as a self-assessment, or by external staff, such as Extension (or other SNAP-Ed implementing) staff, to guide the provision of technical assistance.

The FB-HANA can be used to initiate preparation and planning to promote nutrition and health as well as to supplement existing resources. Many of the food banks who contributed to reliability testing intended to use the results to inform organisational plans. These plans included the development and adoption of nutrition policies, distribution of surveys to staff or clients and initiation of a nutrition advisory board. Extension staff are using the assessments conducted for the reliability phase as baseline measures to evaluate the implementation of policies and practices. Results suggest these evaluations could be completed by Extension or food bank staff, given the strong test-retest reliability for the FB-HANA regardless of assessor type. However, it is worth considering that food bank staff systematically scored their food banks higher on the FB-HANA than Extension staff. Future research, particularly qualitative studies, might consider exploring what contributes to these scoring differences as well as the unique approaches that Extension and food bank staff take in planning PSE initiatives.

Planning for PSE initiatives, regardless of whether they are lead by Extension or food bank staff can be further supported by Feeding America’s Nutrition in Food Banking Toolkit^(43)^ as well as the published case studies of other food banks’ efforts to implement changes in practices^(36,37)^. FB-HANA evaluations and subsequent planning might also support and supplement downstream efforts to assess and improve the consumer nutrition environment in food pantries^(22,23)^. In a Minnesota sample, food banks comprised an average of 50 % of food pantries’ inventories, but this varied from 3 to 99 % in the sample^(44)^, so complementary efforts in both food banks and food pantries are warranted.

A clear insight from the FB-HANA development process was the need for recognition and assessment of who is being served, and by whom, within the charitable food system. At the national level, young adults, women, non-Hispanic Black adults, Hispanic adults, low-income households, adults with a disability and single parents are among those most likely to need the supports of the charitable food system^(4,10)^. However, there is geographic and cultural variation in characteristics of individuals of families seeking support from the charitable food system, therefore food banks and pantries are increasingly striving to reflect the cultural composition and diversity of their respective communities among their staff and volunteers as well as with the food and services offered. The need for community assessment, tailoring to community needs and representation of clientele in decision making are recommended in the Nutrition in Food Banking Toolkit^(43)^. Thus, these considerations were incorporated into numerous strategies in the final FB-HANA. Assessments completed by food bank staff at the initial reliability assessment indicated that over half of food banks identified the diet-related conditions of concern (55 %) or sought feedback about preferred foods (73 %) among individuals served by their member agencies, but few had advisory boards which included current or former recipients of charitable food assistance (18 %; data not shown).

Another important insight from the FB-HANA development was the common practice of food banks connecting to a variety of organisations in the food system and larger community. Though speculative, food banks which have numerous, diverse connections in the community may be able to provide more variety in their food and services as well as have greater capacity to restrict or reject donated goods from a single donor source. Diverse connections may position food banks to be more nimble and adaptable to surges in charitable food reliance, such as the spike in need in 2020 as a result of the COVID-19 pandemic^(10)^. Future climate or public health emergencies may cause similar rapid increases in charitable food system reliance. Food banks may be positioned to adapt to disruptions in the food system by adopting a nutrition policy, which can guide the use of funds to purchase foods. These hypotheses should be tested in future observational studies assessing how food bank characteristics relate to PSE initiatives.

Notably, there are factors upstream of the food bank which can impact the food bank’s ability to promote nutrition and health. For example, national and state-level policies that impact the ability of farmers to sell or donate produce can act as an incentive or deterrent to improving the charitable food system’s quality of foods^(45)^. Gleaning un-harvested foods from farmers’ fields is not commonly reported by food banks^(18)^, but food banks expanding their presence and connection to farmers, gardeners and other community members can help optimise the ability of gleaning to contribute nutritious fresh produce^(39)^. Though challenges to transporting, storing and distributing perishable foods in the charitable food system will need to be taken into consideration^(45–47)^.

Though tools like the FB-HANA and the corresponding instrument for food pantry settings, the Nutrition Environment Food Pantry Assessment Tool^(22)^, have a role in supporting the nutrition and health promotion in the charitable foods system, there are inherent issues related to justice and equity within the system. For many, there is stigma associated with eating donated food, though still healthy and safe, which otherwise may have been discarded^(18)^. Volunteers often far outnumber staff in organisations within the charitable food system^(15)^, which can result in unstable or unpredictable labour. Further, food pantry clientele are often not represented among leaders in the charitable food system^(48)^. This may, in part, explain why results of a recent qualitative study found that stakeholders wrongly perceived the charitable food system as contributing only negligibly to clients’ diets overall^(48)^. In response to these structural inequities, some food banks have shifted to community-hub models where redistributing food is supplemented with additional activities, such as a Speaker’s Bureau which focuses on leadership development and advocacy among food pantry clientele^(36)^, incorporation of a milk bank, policy change advocacy and benefits enrollment support^(49)^. Though the research team incorporated many of these innovations into strategies in the FB-HANA, additional efforts outside of the charitable food system are likely needed to address food access inequities.

### Limitations

The current research study is not without limitations. The sample of food banks who participated in reliability testing for the FB-HANA were limited to four midwestern states. These states may employ policies and practices which are systematically different than those used by food banks in other regions. Fortunately, content validity reviewers had experience with food banking practices in states outside this region, which may have resulted in a greater variety of strategies being added to the final FB-HANA prior to reliability testing. Nonetheless, future studies which draw data from food banks across the USA would be valuable to characterisze practices in different settings and could provide valuable data to assess floor or ceiling effects within the measure. Though the overall FB-HANA scores had evidence of excellent reliability, individual objectives’ reliability estimates were lower. This variation in reliability was likely related to the lack of formal training needed for the FB-HANA and may improve with additional training. One final limitation is that results do not provide insight into how policies and practices may relate to downstream nutrition or health effects among clients of the charitable food system. Additional research will be needed to investigate whether food banking policies and practices translate to outcomes among food pantry clientele.

## Conclusions

Evidence suggests that the FB-HANA is a content valid tool with adequate inter-rater and test-retest reliability that can provide insights on how food banks promote health and nutrition through PSE approaches. Furthermore, the FB-HANA can be used by both food bank and external staff. Early informal results suggested that the FB-HANA can be used to initiate conversations related to policy adoption and practice changes, contributing to the evolution of the charitable food system to better meet needs of clientele by promoting nutrition, health and equity.

## Supplementary material

For supplementary material accompanying this paper visit https://doi.org/10.1017/S1368980023000204. click here to view supplementary material
