# Supplementary material for: Development and evaluation of the Food Bank Health and Nutrition Assessment
Source: Public Health Nutr. 2023 Jan 30;26(4):738–47. doi: 10.1017/S1368980023000204 (PMC10131141; doi:10.1017/S1368980023000204)
Supplement: Supplementary file 1 [file S1368980023000204sup001.docx]

**Supplemental Table: Food Bank Health and Nutrition Assessment (FB-HANA) List of Strategies**

| **Objective 1: Food Bank Identifies & Responds to Nutrition Needs of Communities Served** | | |
| --- | --- | --- |
| 1.1 | Distributes food to groups or geographies identified as having the greatest need for food assistance. | |
| 1.2 | Identifies diet-related health conditions of individuals served by member agencies (i.e., agencies who partner with the food bank to distribute food) through surveys distributed by the food bank or by member agencies. | |
| 1.3 | Items for groups with identified dietary needs (e.g., low-sodium, Heart-Check mark, vegan, gluten-free, Halal, or Kosher, etc.) are labeled in the food bank ordering system. | |
| 1.4 | Shelf-stable ready-to-eat items (e.g., pop-top, easy open lids, and items that require little to no preparation) are easy to identify in the food bank ordering system. | |
| 1.5 | Provides information about, or application support for, food assistance programs (e.g., SNAP, WIC, SFMNP, food pantry lists, etc.) directly to eligible audiences, or by assisting member agencies' efforts. | |
| 1.6 | Provides information about, or application support for, affordable healthcare programs (e.g., Medicaid/Medicare, Affordable Care Act, mental health services, etc.) directly to eligible audiences, or by assisting member agencies' efforts. | |
| **Objective 2: Food Bank Integrates Needs of Diverse Populations** | | |
| 2.1 | Identifies racial and ethnic groups served by member agencies through surveys or secondary sources (e.g., community health needs assessment, member agencies, etc.). | |
|  | 2.1a | Seeks feedback about preferred foods from clients served by member agencies. |
|  | 2.1b | Uses feedback received from clients served by member agencies to seek nutritious, culturally relevant food options for racial and ethnic groups identified. |
| 2.2 | Has a nutrition and/or health equity advisory group. This can include nutrition advisory councils, health equity boards, partner advisory councils, or other groups that specifically address the topics of nutrition and/or health equity. | |
|  | 2.2a | Advisory group includes member(s) who previously or currently make use of food programs offered. |
| 2.3 | Provides nutrition and/or health resources to member agencies in languages spoken by audiences served. | |
| **Objective 3: Food Bank Adopts, Implements, and Shares Nutrition Policy** | | |
| 3.1 | Has a written nutrition policy in place. | |
|  | 3.1a | Policy includes a statement of purpose. |
|  | 3.1b | Policy outlines a rationale for and benefits of the policy. |
|  | 3.1c | Policy outlines food inventory sources. |
|  | 3.1d | Policy outlines foods to increase. |
|  | 3.1e | Policy outlines foods to decrease and/or divert. |
|  | 3.1f | Policy outlines nutrition guidelines for special programs (e.g., school-based food pantries, summer meals, mobile food distributions, etc.). |
| 3.2 | Food bank seeks input on nutrition policy, and any substantive updates, from clients served by member agencies. | |
| 3.3 | Nutrition policy is reviewed annually, and updates are made (by leadership, nutrition advisory group, board of directors, etc.) as needed. | |
| 3.4 | Nutrition policy is shared publicly. At a minimum, a summary of the policy is physically posted at the food bank and the full policy is easy to find on the food bank's website. | |
| 3.5 | Nutrition policy training is included in new staff member, board member, volunteer, and member agency onboarding. The type and amount of training provided can be role specific. | |
| 3.6 | The nutrition policy is reviewed with staff members, board members, volunteers, and member agencies at least annually, or when the policy is updated. | |
| 3.7 | Donors or industry partners are informed of the nutrition policy. | |
| **Objective 4: Food Bank Provides Nutrition Education Training & Nutrition Education Resources** | | |
| 4.1 | Nutrition education training is provided to staff members during onboarding and on an annual basis. | |
| 4.2 | Nutrition education training is provided to volunteers during onboarding and on an annual basis. | |
| 4.3 | Nutrition education training is provided to board members during onboarding and on an annual basis. | |
| 4.4 | Nutrition education training is provided to member agencies during onboarding and on an annual basis. | |
| 4.5 | Nutrition education resources (e.g., flyers, fact sheets, etc.) are made available to member agencies. | |
| 4.6 | Nutrition education resources (e.g., flyers, recipe cards, fact sheets, etc.) are made available to donors, industry partners, and community stakeholders. | |
| 4.7 | Nutrition education (e.g., cooking demonstrations, in-person education, recordings, etc.) is provided to clients of member agencies, directly or through partnership. | |
| **Objective 5: Food Bank Fosters a Variety of External Partnerships** | | |
| 5.1 | Has active partnership(s) with healthcare organizations (e.g., hospitals, clinics, etc.). | |
| 5.2 | Has active partnership(s) with public health organizations (e.g., public health department, WIC, SNAP-Education, etc.). | |
| 5.3 | Has active partnership(s) with colleges or universities to support health initiatives. | |
| 5.4 | Has active partnership(s) with gleaning sources of nutritious food (i.e., local gardeners, farmers, etc.). | |
| 5.5 | Has active partnership(s) with population-specific or culture-specific groups/organizations (e.g., Immigrant and refugee groups, non-native English-speaking groups, etc.). | |
| 5.6 | Participates in state, regional, or local health or food system/policy coalitions. | |
| 5.7 | Annually recognizes exceptional community partnerships (i.e., non-member agencies) for health promoting practices or initiatives. | |
| **Objective 6: Food Bank Prioritizes Health in Internal Operations & with Member Agencies** | | |
| 6.1 | Has line item in the budget to purchase food. | |
|  | 6.1a | A portion of the budget is earmarked for nutritious foods. |
| 6.2 | Has line item in the budget to support member agency adoption or maintenance of health promotion practices. | |
| 6.3 | Seeks donations, grants, or hosts fundraisers for health initiatives and earmarks those funds for these initiatives. | |
| 6.4 | Seeks or designates funds to subsidize nutritious items for member agencies. | |
| 6.5 | Implements pricing strategies to promote nutritious food items to member agencies. (e.g., makes produce free, prices low sodium soups lower than regular soups, etc.). | |
| 6.6 | Encourages member agencies to use client choice distribution models. This can include requiring, incentivizing, or providing training on client choice models. | |
| 6.7 | Makes recipes available to member agencies that include nutritious foods. | |
|  | 6.7a | Recipes made available include those that require minimal cooking equipment. |
|  | 6.7b | Recipes made available consider accessibility for a variety of audiences (e.g., plain language, images, fewer than ten ingredients, minimal or no abbreviations, culturally-relevant ingredients, etc.). |
| 6.8 | Annually recognizes exceptional member agencies or volunteers for their health promoting practices or initiatives. | |
| **Objective 7: Food Bank Considers Nutritional Quality of Commodity, Purchased, & Donated Foods** | | |
| 7.1 | Has adopted a formal system for ranking or classifying nutrition content of foods (e.g., HER Nutrition Guidelines, SWAP, MyPlate, Nourish, etc.). | |
|  | 7.1a | Which system has the food bank adopted? |
|  |  | HER Nutrition Guidelines |
|  |  | SWAP |
|  |  | MyPlate |
|  |  | Nourish |
|  |  | Other, please describe |
|  | 7.1b | Applies ranking system to government foods. |
|  | 7.1c | Applies ranking system to purchased foods. |
|  | 7.1d | Applies ranking system to donated foods. |
|  | 7.1e | Nutritious items are easy to identify in the food bank ordering system. |
| 7.2 | Has strategy in place to divert non-nutritious donated food items (e.g., providing food to composting facilities or farmers, as allowable by local and state policies). | |
| 7.3 | Does not accept bulk donations for non-nutritious foods (e.g., sodas, candy, etc.). | |
| 7.4 | Food drives hosted by the food bank emphasize nutritious foods. | |
| **Objective 8: Food Bank Models Nutrition Promotion Practices to Member Agencies** | | |
| 8.1 | Displays nutritious items more prominently (i.e., before or in front of) than less nutritious options. | |
| 8.2 | Nutritious foods are easily accessible. | |
| 8.3 | At least one nutritious item is on display as part of a bundle (i.e., meal kit, recipe, food pairing, etc.). | |
| 8.4 | Colorful posters, shelf tags/shelf-talkers, signage, banners, and other healthy eating materials are visible. | |
| 8.5 | Fresh produce is free of severe damage and signs of excessive over-ripening. | |
| 8.6 | Keeps spaces clean and well-organized. | |
